# Playing Brains: The Ethical Challenges Posed by Silicon Sentience and Hybrid Intelligence in DishBrain

**DOI:** 10.1007/s11948-023-00457-x

**Published:** 2023-10-26

**Authors:** Stephen R. Milford, David Shaw, Georg Starke

**Affiliations:** 1 Department of Theology, North-West University, Potchefstroom, South Africa; 2 Institute for Biomedical Ethics, Basel University, Basel, Switzerland; 3 Maastricht University, Maastricht, Netherlands; 4 College of Humanities, École Polytechnique Fédérale de Lausanne, Lausanne, Switzerland; 5 Institute for History and Ethics of Medicine, Technical University of Munich, Munich, Germany; 6 Munich School of Philosophy, Munich, Germany

**Keywords:** DishBrain, Consciousness, Brain organoid ethics, Free energy principle, AI ethics, Synthetic biological intelligence

## Abstract

The convergence of human and artificial intelligence is currently receiving considerable scholarly attention. Much debate about the resulting *Hybrid Minds* focuses on the integration of artificial intelligence into the human brain through intelligent brain-computer interfaces as they enter clinical use. In this contribution we discuss a complementary development: the integration of a functional in vitro network of human neurons into an *in silico* computing environment.

To do so, we draw on a recent experiment reporting the creation of silico-biological intelligence as a case study (Kagan et al., 2022b). In this experiment, multielectrode arrays were plated with stem cell-derived human neurons, creating a system which the authors call *DishBrain*. By embedding the system into a virtual game-world, neural clusters were able to receive electrical input signals from the game-world and to respond appropriately with output signals from pre-assigned motor regions. Using this design, the authors demonstrate how the *DishBrain* self-organises and successfully learns to play the computer game ‘Pong’, exhibiting ‘sentient’ and intelligent behaviour in its virtual environment.

The creation of such hybrid, silico-biological intelligence raises numerous ethical challenges. Following the neuroscientific framework embraced by the authors themselves, we discuss the arising ethical challenges in the context of Karl Friston’s Free Energy Principle, focusing on the risk of creating synthetic phenomenology. Following the *DishBrain’s* creator’s neuroscientific assumptions, we highlight how DishBrain’s design may risk bringing about artificial suffering and argue for a congruently cautious approach to such synthetic biological intelligence.

## Introduction

In a widely reported study, Brett Kagan and his team created lab-grown human neuron clusters that learned how to play a simple computer game. To achieve this, the team cultured neurons from rodents and humans and embedded them using electronic circuitry into a simulated game-world similar to ‘pong’ (a popular computer game in the late 1970’s). Their research and findings (Kagan et al., 2022b) make for astounding reading, not only for their technical sophistication, but also for their possible applications, and the implications for bioethics.^1^

In this paper, we analyse the ethical challenges posed by synthetic biological intelligence (SBI) such as the system created by Kagan et al. Our approach is different to that taken by the system’s creators. While we agree that the ethical permissibility of brain organoids ultimately hinges on their capacity to have phenomenally conscious experiences, we further think it convincing to adhere to the neuroscientific framework of Kagan et al. themselves – predictive processing based on the free energy principle – when analysing their system with view to questions of consciousness. However, unlike Kagan et al., following this intuition, we defend the precautionary principle with view to a theory of consciousness that is in line with the free energy principle (Solms, 2019; Solms & Friston, 2018). We show that while *DishBrain* itself may not be phenomenally conscious, future refinements of such a system very possibly may be. Drawing on Thomas Metzinger’s argument from artificial suffering, we therefore argue for a congruently cautious approach to SBI.

We begin our paper with a brief overview of Kagan et. al.’s research and the claim that their system reached a form of sentience. This introduction is followed by a discussion on the neuroethical challenges posed by brain organoids, as well as a discussion on consciousness in terms of its definition and measures, its relation to the free energy principle, and the ensuing moral implications. Our final section will consider the precautionary principle before concluding this paper with our recommendations for the near future development of SBI.

## An Introduction to Playing Brains

Kagan and his team created a system they call *DishBrain*: a functional in vitro network of neural cells integrated into an in-silico computing environment. To do so, the team plated multielectrode arrays with approximately 10^6^ cortical cells each, sourced from embryonic rodent and human induced pluripotent stem cells (hiPSCs; (Kagan et al., 2022b).^2^ Within these arrays, a sensory area and motor region were predefined. Electric signals from the game-world were electrically transmitted to the sensory region of the neural networks while the output from the motor regions of the networks was fed back into the game-world. Depending on the read-out signal, the position of a virtual paddle was adjusted, reflecting a virtual ball, and the success of each game run was in turn fed back to the sensory system through providing or withholding electric stimulation. In doing so, the authors claim that BrainDish harnesses ‘the computational power of living neurons to create synthetic biological intelligence (SBI)’ (Kagan et al., 2022b, p. 3952). Using the inherent property of neurons to share electrical (synaptic) activity with each other, DishBrain can stimulate and record the synaptic ‘language’ to link silicon and biological neural networks (BNN). The result is a closed feedback system of neurons receiving stimulation from a simulated game-world and responding to those stimulations accordingly.

By integrating lab-grown neurons and multielectrode arrays, the authors contribute to the development of silico-biological intelligence, providing a hybrid of neurons and technical artifact that some believe may ultimately outperform purely in silico artificial intelligence. In line with Buchanan (2018), Kagan et al. note that silicon-alone hardware has serious performance constraints that may limit the development of artificial general intelligence (AGI). However, considering recent biological developments in culturing organoids, they point to the possibility of SBI arriving before AGI. Kagan et al.’s research is, therefore, framed in a way that appears to aim at developing SBI as a step towards AGI.

One element commonly discussed in the context of AGI is that systems may achieve sentience, with the debate sparked by Google’s Language Model for Dialogue Applications (LaMDA) being one prominent example (Curtis & Savulescu, 2022). In comparison, Kagan et al. define sentience rather minimally “as being ‘responsive to sensory impressions’ through adaptive internal processes” (Kagan et al., 2022b, p. 3952, citing Friston et al. (2020).^3^ In their paper, Kagan et al. argue that two interrelated processes are needed to achieve sentience. The first is the system’s ability to learn how external states influence internal states through perception. The second is that a system must use its sensory states to infer when it should adopt a particular behaviour. In other words, the system must be able to perceive its environment and adopt responsive behaviours. Since *DishBrain* meets both conditions, the paper concludes that in their setting “in vitro cortical neurons can self-organize and display intelligent and sentient behavior when embodied in a simulated game-world” (Kagan et al., 2022b, pp. 3966). This bold claim concerning an artificial system using human neurons to display sentient behaviour raises significant ethical questions.

Yet, Kagan et al.’s ethics’ statement is surprisingly short. It states that their experiments were conducted in accordance with the Australian National Statement on Ethical Conduct in Human Research (2007) as well as the Australian Code for the Care and Use of Animals for Scientific Purposes (2013). It notes that successful applications were made to their respective institutions but is very meagre on its description of the key ethical issues raised by their research. Notwithstanding this, they have provided a more thorough ethical debate of their work in a later open peer commentary (Kagan et al., 2022a) in which they reply to an ethical debate on brain organoids (Sawai et al., 2022).

In this comment, Kagan et al. stress the difference between consciousness and sentience, arguing for vastly distinct implications resulting from phenomenal self-consciousness on the one hand, and the mere ability to respond to sensory stimuli on the other hand (Kagan et al., 2022a). This move appears shrewd and is well-motivated by their reference to studies in blindsight patients. Furthermore, it is also consistent with the original DishBrain paper, which embraces a minimal definition of sentience built on two conditions:

Firstly, the system must learn how external states influence internal states – via perception – and how internal states influence external states – via action. Secondly, the system must infer from its sensory states when it should adopt a particular behavior. In short, it must be able to predict how its actions will influence the environment. (Kagan et al., 2022b, p. 3953)

Following this semantic clarification, the authors then proceed to argue against Sawai et al. and against employing a precautionary principle with a view to brain organoids. They do so by focusing on the integrated information theory (IIT) of consciousness stressed by Sawai et al. and colleagues. In lieu of empirical evidence of consciousness as predicted by this theory in current brain organoid research, Kagan et al. argue that it would be undesirable, and potentially even unethical, to ban such research given its potential benefits (2022a).

## Neuroethical Challenges Posed by Brain Organoids

The field of cerebral organoid research is young but developing rapidly. A key development in this field has been the successful use of human induced pluripotent stem cells (hiPSCs; (Lancaster & Knoblich, 2014), rendering the controversial collection of embryonal stem cells obsolete and opening new research avenues. Just recently, Neanderthal DNA was introduced to hiPSCs using CRISPR from which cortical organoids were cultured (Trujillo et al., 2021), while other experiments have introduced human cortical organoids into the brains of other animals (Mansour et al., 2018) forming neural chimera states (Calim et al., 2020; Farahany et al., 2018, Khaleghi et al., 2019). In fact, Kagan’s team is not alone in attempting to embody neural organoids. Potential advantages of such research are clear: from helping us understand evolution (Dannemann & Gallego Romero, 2021), Zika (Qian et al., 2016), to Alzheimer’s (Chen et al., 2021) and autism spectrum disorders (Ilieva et al., 2018), the research promises to reduce human suffering caused by neurological and psychiatric disorders. In fact, so large seem the promises of the field that it may be considered unethical to abandon it (Farahany et al., 2018).

Yet, at the same time, there are a host of ethical concerns (Schneider et al., 2023). One of the most fundamental risks is artificially creating phenomenally conscious systems capable of suffering (Sawai et al., 2022). Later parts of this paper will focus on this very risk. However, there are also other important ethical concerns. Farahany et al. (2018) list several, noting the blurring of human-animal distinctions; the challenge that brains dying in dishes poses to our understanding of life and death; issues of consent, stewardship, and ownership; as well as how we handle post-research brain tissue. All this is not to mention the legalities in terms of data rights, how research is shared, and who benefits from the discoveries. Considering that the field is still young, with first evidence of cerebral organoids dating to 2008 (Eiraku et al., 2008), there remain few robust ethical guidelines.

Currently, research on brain organoids is conducted in accordance with standard rules of the international scientific community, making use of local ethics committees. Recently this has attracted some attention and some moves have been made to develop a more specific framework. This includes the BRAIN Initiative from the National Institute of Health which have produced some guidelines (Bianchi et al., 2018; Greely et al., 2018; Ramos et al., 2018) as well as the Human Brain Project which has an ethics component (Salles et al., 2019). Nevertheless there is still consensus that far more needs to be done (Farahany et al., 2018). To use Rommelfanger’s words, ‘we need neuroethicists present before the Holy S**T moments in neuroscience’ (2019).

As we cannot cover the broad ethical debate about brain organoids in our paper, we will focus here on points that are particularly relevant to the case study at hand. Until recently, cerebral organoids lacked either inputs or outputs, and for this reason some argued that they were of diminished moral concern (Lavazza, 2021; Lavazza & Massimini, 2018). This, however, has been seriously disputed. There is ample evidence that hiPSCs are synaptically active and connected (Paşca et al., 2015; Pașca, 2018). After just two and a half months of development, cortical neurons resemble mid-fetal prenatal brain development (19–24 post-conception weeks), while ten month-old human cortical organoids develop according to specific genetic programmes and manifest complex brain activity including synaptic firing rates of 3 to 4 per second, as well as the kinds of gamma, alpha, and delta waves that are the hallmark of a vital human brain (Lavazza, 2021; Lavazza & Massimini, 2018; Lavazza & Pizzetti, 2020). In fact, whole-brain organoids, those not developed with a specific focus such as forebrain or cerebellum, show electrical activity very similar to that of preterm infant brains (Lavazza & Pizzetti, 2020).

One challenge that *DishBrain* seems to have overcome is that lab-grown organoids suffer from a developmental plateau. Therefore, while it may be argued that they have not developed an organoid per se (and are not aiming for one), their experiment seems to successfully overcome the challenge of a developmental plateau. In fact, *DishBrain* focuses precisely on inputs and outputs. It does this by predefining input and output regions on the multiarray, allowing the cultured neurons to self-organize into sensory and motor regions, capable of receiving input and providing relevant output signals. What is pertinent here is that Kagan et al. note that this results in learning. Learning is arguably evidence of development. That is to say, Kagan et al. noted that at the beginning of their experiment neuronal synaptic firing was sporadic, chaotic, and random. However, after receiving inputs and providing opportunities for outputs that further impact future inputs, the neural synaptic firing became organised, co-ordinated, and directed toward a particular goal.

In other words, there is evidence of learning: *DishBrain* appears to be able to adapt to its environment by making increasingly correct predictions that result in action. Its creators have framed and explained this learning in the context of Karl Friston’s so-called *Free Energy Principle*, which provides an important angle for discussing its potential for both sentience and consciousness. Yet, before we can turn to the Free Energy Principle, a brief clarification on the search for neural correlates of consciousness is in order, situating *DishBrain* in the wider landscape of ongoing research.

## Consciousness: Terms and Measures

The question of consciousness is at the core of myriad research efforts in philosophy. Particularly challenging remains the so-called hard problem of consciousness, i.e. the difficulty of explaining the experiential, phenomenological dimension of conscious experience (Chalmers, 1995). This hard problem can be distinguished from the (comparatively) “easy” problem of explaining functional and behavioural aspects of consciousness. Modern neuroscience contributes to both endeavours by seeking specific neural correlates of consciousness (NCC). In this context, it is key to be clear in one’s terminology. For example, while consciousness is often associated with self-awareness and attention directed towards an object, we sometimes also talk about consciousness to denote that someone is awake and not, for example, in a coma.

One classical distinction, therefore, distinguishes between *creature consciousness*, describing the fact that an animal is awake and possibly able to make experiences, and *state consciousness*, denoting what it is like to have a specific experience (Block, 1995; Nagel, 1974; Rosenthal, 1997). In the context of our paper and following other research on the ethics of brain organoids (Sawai et al., 2022), we are primarily interested in the latter. The question is: how could one determine whether *DishBrain* has gained creature or even state consciousness?

To determine this question, it seems valuable to briefly look at the methods which serve to investigate creature and state consciousness. Of particular interest to the case at hand are brain-based indexes of (creature) consciousness that are independent of sensory processing or motor outputs (Lavazza, 2021; Lavazza & Pizzetti, 2020). Using electroencephalography (EEG) or functional neuroimaging paradigms (often based on perturbational approaches), some argue that it is possible to identify not only the presence, but the level of consciousness in unresponsive patients (Gosseries et al., 2014). A prime example is Casali et al.’s Perturbational Complexity Index (PCI; Casali et al., 2013). PCI is based on the theory that consciousness is dependent on a brain’s ability to support complex activity patterns distributed among interacting cortical areas that are also differentiated in space and time. PCI is calculated by first perturbing the cortex with transcranial magnetic stimulation (TMS) and compressing the spatiotemporal pattern of electrocortical responses to measure their complexity (information). According to Casali et al., the PCI reliably decerns the level of consciousness in patients who were wakeful, asleep, in anaesthesia, as well as those who emerged from a coma or recovered a minimal level of consciousness.

Would it be possible to apply such a metric to *DishBrain* so as to ascertain its level of creature consciousness? PCI readings reach a high value (indicating high levels of consciousness) only where the initial disturbance (perturbation) is transmitted to a large network of neuronal elements that react in differentiated ways (Lavazza & Pizzetti, 2020). If this metric were to be applied to *DishBrain*, however, it is unlikely that there would be a large enough network of neuronal elements to react in differentiated ways. For now, the number of neurons is simply too small. It is important, however, that one keeps in mind that many metrics of consciousness do not apply uniformly across the human population. Infants, for example, provide different scores than adults when subjected to similar testing (Farahany et al., 2018). Consequently, even if one could determine a PCI score for *DishBrain*, it would be entirely unclear how to interpret it.

Even more intricate than measuring creature consciousness remain attempts to tackle the hard problem of state consciousness and to measure the presence of phenomenal consciousness in humans. In fact, many prominent neuroscientists hold that we can only determine the presence of phenomenal consciousness by relying on verbal self-reports, barring its ascription to non-human animals (LeDoux, 2015).^4^ Are we, therefore, justified in assuming the absence of phenomenal consciousness in non-responsive humans? We think not. Think for example of patients with brain injuries, suffering from completely locked-in syndrome, or those slowly waking from a comma. In these cases the opportunity to speak to the conscious subject is limited, as is the availability of empirical evidence for motor responses and sensory processing. Is *DishBrain* such a patient? If so (or not) how can we tell?

How one answers this question depends largely on the theory of consciousness to which one subscribes. While the search for a comprehensive, neuroscientifically grounded theory of consciousness is ongoing (Seth & Bayne, 2022), there are certain points that seem uncontroversial among the majority of theories. First, it seems highly plausible that certain brains structures and processes have a privileged relationship with subjective phenomenological experiences. For example, the neurons in thalamocortical circuits seem essential in many models, while those in the cerebellar less so, despite their huge numbers. In other words, it seems that it is not merely the number of neurons that are important to bringing about consciousness, but their type and structure. Second, phenomenal consciousness is directed towards an object; it is intentional. For instance, one may ask what it is like to see a particular colour, or what it is like to hear a particular piece of music. Even if the piece of music consists of silence, as in John Cage’s 4’ 33”, the phenomenal experience is directed towards something, namely the absence of sound. In either case, phenomenal consciousness appears to require the possibility of sensory input.

How exactly consciousness is brought about on a neural level differs largely among major theoretical strands, such as, integrated information theory; higher-order theories; global workspace theories; or predictive processing theories (Seth & Bayne, 2022). When investigating a phenomenon like consciousness, one’s assumptions about consciousness greatly influence one’s predictions about where to find it. For example, a theory based on higher-order thinking would make different predictions about the types of systems that support consciousness than would a theory emphasising attentional processes (Shepherd, 2018). To avoid imposing our own perspective on consciousness on someone else’s work, we suggest examining the question of consciousness with view to *DishBrain* by adhering to the neuroscientific theory to which its authors themselves subscribe: a theory of predictive processing based on the Free Energy Principle (Kagan et al., 2022a, p. 3).

## Consciousness and the Free Energy Principle

One of the main contributions of Kagan et al.’s paper lies, according to its authors, in their development of an artificially created system that supports one of the most influential neuroscientific theories of the last decade, namely that complex neural systems have an innate tendency to minimize their free energy (Kagan et al., 2022b). This theory, formulated first by Karl Friston, offers a ‘unified brain theory’ in the eyes of its proponents and postulates that the brain – as well as other biological systems – minimize the long-term average of surprise associated with sensory exchanges with the world (Friston, 2010). This surprise can be formulated mathematically as ‘free energy’ and is, according to this theory, minimized by biological organisms in an effort to resist the natural tendency to disorder.

Free energy in the sense of surprise presupposes predictions about internal or external sensory inputs. Surprise ensues when the brain’s predictions, based on an internal generative model of the world, do not match sensory input. According to the free energy principle, organisms aim to minimize surprise, and can do so in several ways. They can either change and update the predictions themselves (top-down), so that they match the sensory input, or they can change their environment to change the sensory inputs (bottom-up) so that it matches the prediction (Seth & Bayne, 2022). Due to the intricate connection of predicting and acting, this process is also called active inference (Friston et al., 2017).

Based on this model, the free energy principle attempts to provide a unified theory that seeks to explain how we perceive, learn, and act under conditions of uncertainty. As Andy Clark put it eloquently, our brains ‘surf’ on this uncertainty, staying ahead of the ever changing wave: ‘To deal rapidly and fluently with an uncertain and noisy world, brains like ours have become masters of prediction – surfing the waves of noisy and ambiguous sensory stimulation by, in effect, trying to stay just ahead of the place where the wave is breaking’ (Clark, 2016, p. xiv). In this sense, perception can be understood as a process which minimizes free energy ‘with respect to synaptic activity (perceptual inference), efficacy (learning and memory) and gain (attention and salience)’ (Friston, 2010). Learning, in turn, may be seen as optimizing the connections within a multi-layered hierarchical model in which predictions about predictions are constantly updated to minimize surprise (Friston, 2010). Action, finally, can be interpreted as resulting from a minimization of free energy with a view to the environment or even our own body: if movement is seen as adapting to predictions concerning interoceptive sensory input, the free energy principle can provide a simple model of motor control.

While the free energy principle was originally not developed as a theory of consciousness (Seth & Bayne, 2022), there are many prominent attempts to make its reasoning fruitful for the study of consciousness (Clark, 2016; Solms, 2019; Solms & Friston, 2018). One way of doing so is in equating phenomenological consciousness as arising directly from predictive functioning, where it provides an adaptive advantage for survival in novel environments. As Solms puts it, ‘deviation away from a homeostatic settling point (increasing uncertainty) is felt as unpleasure, and returning toward it (decreasing uncertainty) is felt as pleasure’ (Solms, 2019; cf. Solms & Friston, 2018). One may, therefore, say that there is some phenomenological experience, some *what-it-is-likeness* connected to making correct or incorrect predictions.

Free energy-based theories of consciousness also embrace a notion of intentionality in the sense cited above, i.e., that phenomenal consciousness is directed towards something:

The distinction between interoceptive and exteroceptive precision is central to this argument. If brains are sympathetic organs of inference, assimilating exteroceptive (sensory/motor) and interoceptive (vegetative) data through prediction, then their respective precision is about something. (Solms, 2019, citing Brentano 1874)

Yet, not all predictive processes give rise to consciousness (Solms & Friston, 2018). In fact, phenomenal consciousness seems to require some degree of (hierarchical) complexity in a system, which has evolved over time. What may a free energy-based theory of phenomenal consciousness predict with regard to *DishBrain*? The neuron-plated electrodes certainly fulfill the first condition of integrating predictions about external (the position of the ‘pong’ ball) as well as internal states (the predictable electric feedback based on a game’s success). Yet, the limited complexity of 10^6^ self-organized neurons in *DishBrain*, as compared to a human brain with almost 10^11^ neurons, renders phenomenal consciousness on these plates questionable. This uncertainty raises the question: what would be the moral consequences of a conscious synthetic biological intelligence?

## The Moral Implications of Consciousness

It is commonly held that consciousness is of intrinsic value (Gibert & Martin, 2022; Lavazza, 2021; Lavazza & Massimini, 2018), resulting in immediate moral claims by phenomenally conscious entities. Kriegel, for instance, has surveyed a number of different research areas in which the question of the value of consciousness comes into play. He considers a range of research avenues, from epistemic and ethical to aesthetic. Ultimately, he concludes that while there are serious disagreements as to why consciousness has value, it is generally agreed that it does (Kriegel, 2019). As such, the why is not of primary importance, merely that consciousness is an attribute of persons that is of fundamental significance and cannot be ignored. In like fashion, Lavazza argues:

To sum up, it is conceptually and empirically possible to evaluate the basic conditions for attributing a moral status to human cerebral organoids grown in the laboratory. …The presence of consciousness in a human brain, even if in a form inferior to that of a healthy adult individual, constitutes in fact the conceptual and empirical presupposition for the attribution of a moral status. (Lavazza, 2021, p. 6)

However, some hold that phenomenal consciousness in itself does not imply strong moral claims against killing or harming conscious entities. Instead, there is a suggestion that one should focus ‘on the kinds of biopsychological architecture likely to support conscious processes of moral interest’ (Shepherd, 2018, p. 611). According to this view, consciousness contributes to the psychological architecture of a subject that may experience certain phenomena (such as pain, pleasure, higher-order thinking etc.) because they are conscious (Shepherd, 2017, 2018). These experiences, so Shepherd claims, are ultimately significant, whereas consciousness in itself may be neither valuable nor non-valuable in itself. Consciousness thus understood is relegate to the role of an enabler, a determinable property that may enable positive experiences that are valuable because of their specific phenomenal characters.

How does this translate to the case of the DishBrain? If we take its creators at their word and subscribe to a theory of consciousness in line with their neuroscientific theory built on the free energy principle, the difference between consciousness as value in itself, and consciousness as enabler for moral experiences would not matter here. As we have seen, phenomenal consciousness in the theory suggested by Solms and Friston is constructed around pleasure and unpleasure – a dichotomy that is of immediate moral relevance and arguably at the very heart of much utilitarian theory building (Gere, 2017). Consequently, the question of consciousness may be reformulated as follows: is *DishBrain* capable of having morally relevant experiences?

The question is highly pertinent. When the Warnock Committee conferred moral status to embryos after 14 days old, they did so not on the basis of a high level of consciousness, but on the potential for the embryo to feel pain at some point after two weeks. Here the committee assigned moral status and value (and therefore rights) on the basis of three elements: (1) a human origin, (2) the potential ability to feel pain and (3) the potential to generate an individual human being (Lavazza, 2021).

It is arguable that *DishBrain* satisfies two of these criteria. Using human neurons gives this experiment a human origin, satisfying the first condition. Second, *DishBrain* can receive stimulus from its environment and respond appropriately to them, arguably based on active inference. While the Warnock Committee focused on pain and suffering as negative non-valuable experiences, this does not exclude the moral relevance of other negative and positive experiences. Let us summarize that, based on the very theory its creators employ, it may well ensue that the system experiences pleasure and unpleasure, resulting from their minimization of prediction errors. We return to the importance of negative valence for artificial suffering in the following section.

## *DishBrain* and the Precautionary Principle

Should we ban research developing silico-biological intelligence like *DishBrain*? This is the conclusion its creators seem to fear. In their ethical commentary, they point to the numerous potential benefits of developing brain organoids and emphatically warn against a slippery-slope rhetoric, barring future progress: ‘Before discussions about the ethical line between what should be ‘permissible and impermissible’ within research on brain organoids, there is substantial work required for an objective approach driven by constructive goals. At all costs, we should avoid a slippery slope rhetoric at such an early stage of research’ (Kagan et al., 2022a). In contrast to these moderately worded reassurances, stands a rather sensationalist communication published on *Medium* by Cortical Labs, the institution who developed *DishBrain*. Here, the claim is much bolder: “[W]e don’t know what we’re making, because nothing like this has ever existed before. An entirely new mode of being. A fusion of silicon and neuron. A native to the digital world lit with the promethean fire of the human mind (Cortical Labs, 2021).

Of course, press releases need to be read with a grain of salt, as they tend to exaggerate and use attention grabbing wording. Recent scholarly literature has therefore rightfully addressed the problem of communicating scientific findings to the general public with view to brain organoids in general (Bassil, 2023) and the *DishBrain* in particular (Balci et al., 2023; Rommelfanger et al., 2023). Of greater interest to us though is the *DishBrain* creators’ statement that they do not know *what* they are making, which seems to be plausible independently of their press release. What are we to make of such natives to the digital world then? What kind of moral status should be granted to these new entities, by whom and on what basis?

It is precisely such uncertainties which have motivated ethicists to call for caution in research on brain organoids and SBI (Sawai et al., 2022). Going a step further, the philosopher Thomas Metzinger has even called for a global moratorium on the development of synthetic phenomenology, i.e. on creating artificial beings that are potentially able to have a morally relevant experiences in the sense of artificial suffering (Metzinger, 2021).^5^ Metzinger bases his argument on four conditions, which he deems necessary for conscious suffering. An entity needs to be (1) capable of conscious experiences, (2) possess a phenomenal self-model, (3) have subjective preferences that can be thwarted, resulting in a state of negative valence, and (4) this state needs to be transparent. Let’s look at these four conditions with view to the *DishBrain* experiment in detail.

A precise technical formulation of the first condition remains elusive as long as we do not agree on a theory of consciousness. However, as argued in detail above, a theory of consciousness that is commensurate with the *DishBrain* creators’ convictions concerning the Free Energy Principle would be in line with phenomenally conscious states in a sufficiently complex *DishBrain*. Therefore, the question remains whether the other three conditions could in principle be met by *DishBrain*. For, as Metzinger puts it, “the essence of suffering lies in the fact that a conscious system **forced to identify** with a **state of negative valence** and is **unable to break this identification** or to functionally detach itself from the representational content in question” (Metzinger, 2021, p. 7 highlights by the authors).

This claim comprises what Metzinger sees as further necessary conditions of suffering. First, assuming we can attribute *some* form of consciousness to an artificial entity such as *DishBrain*, it would need to have a phenomenal self-model (PSM) (Metzinger, 2021). In brief, the PSM is required for an entity to identify with an experience, i.e. here, to experience something as their *own* suffering. This condition is crucial as there is empirical evidence that not all phenomenal experience necessarily includes such a form of ownership of experience (Blanke & Metzinger, 2009; Gamma & Metzinger, 2021; Metzinger, 2020). Whether *DishBrain*, as described by Kagan et al., commands such PSM is an open question. However, given that from an evolutionary angle the PSM seems to be closely linked to an organism’s capability of adapting to changing environments (Metzinger, 2021) and that such adaptation driven by minimizing *Free Energy* is at the core of the *DishBrain* experiment, it does not seem entirely unreasonable to at least consider attributing a rudimentary PSM to the system.

The third and fourth condition for artificial suffering according to Metzinger demand that a phenomenal experience has negative valence and that the entity in question cannot distance itself from said experience, i.e. that the experience is transparent, by making “their representational content appear as irrevocably real, as something the existence of which you cannot doubt” (Metzinger, 2021, p.11). As discussed above in detail, the free energy principle entails that prediction errors entail a negative valence. But could *DishBrain* distance itself from such an experience? There is ongoing AI research that attempts to computationally implement a function for such distancing (Agarwal & Edelman, 2020). Yet, since such a model increases the computational load of a model, it seems to have rarely evolved in natural processes. *DishBrain’s* reliance on human neurons does therefore not provide any reason to assume non-transparent phenomenal experiences.

## Conclusion

In conclusion, the *DishBrain* experiment does not provide clear evidence for artificial suffering. Yet, based on the authors’ own conceptual commitments, it is also not incommensurate with the conditions of artificial suffering as formulated by Metzinger. So how should we act from an ethical perspective under such uncertainty? The correct course of action will, of course, depend on one’s ethical convictions. Responding to Metzinger’s argument, there are critics on both sides, arguing that his demands are either impractical and too far reaching (Blackshaw, 2023) or insufficient, as we should potentially also award non-sentient forms of AI moral standing (Ladak, 2023).

In our view, a prudent approach would embrace the precautionary principle and avoid developing artificial entities that may be capable of morally relevant forms of conscious experiences altogether (see also Hildt, 2023). While a moratorium on all related research may be a step too far, we feel that at the very least we should have “discussions about the ethical line between what should be ‘permissible and impermissible’ within research on brain organoids” (Kagan et al., 2022a). These discussion should take place now. They should be aimed at developing an agreed understanding of consciousness, sentience and related phenomena as well as agreed forms of measuring these phenomena. To enable such discussions more conceptual as well as empirical research is needed that brings together expertise from philosophy, computer science and neuroscience.

It is possible that during these discussions researchers may accept certain forms of artificial suffering, similarly to the way in which suffering of non-human animals is accepted in certain forms of research. Nevertheless, until such questions are addressed, and there is some consensus as to what consciousness entails, how it may be measured, and the degree of suffering we are prepared to accept for such entities, research like *DishBrain* should be strictly curtailed. There should be limitations on the number and types of neurons employed in this research, on its experimental applications, and its duration. Failure to do so may result in extensive artificial suffering.

## Footnotes

1 The increasing integration of intelligent neurotechnology into the human brain results in what some have called a hybrid mind (Soekadar et al., 2021). Much ethical debate investigates the use of intelligent neurotechnology in the human brain (Gilbert et al., 2019; Klein et al., 2016; Muñoz et al., 2020), however, the integration of human neurobiology into AI has so far received relatively little attention.

2 Interestingly, the human cell group performed significantly worse when first introduced to the embodied environment. However, this trend later reversed, with the human cell group outperforming all other control groups in all measures. This includes demonstrating considerably increased mean synaptic firing rate, learning, as well as substantially longer average rally lengths than cultures of mice cortical cells. The authors, therefore, conclude that their findings support ‘the hypothesis that human neurons have superior information-processing capacity over rodent neurons’ (Kagan et al., 2022b, p. 3966). As the number of cells were comparable between the human and rodent control group, as was the embodied environment, this superiority appears to be on a cellular level. The authors speculate that this is due to the denser and longer dendritic trees in human neurons that yield different input-output properties.

3 The authors‘ use of “sentience“ sparked intense debate concerning semantics and public communication (Balci et al., 2023; Kagan et al., 2023). To be charitable, we adhere to the original authors‘ use of “sentience” here (Balci et al., 2023, Kagan et al., 2023).

4 Some even go further and argue for the inability to prove consciousness save for our first-hand experience of consciousness. Here, we side with authors who argues that since others speak, act, have similar brains, and share a similar evolutionary heritage to us, it is sensible to assume they are conscious even before their saying so. The onus lies to prove otherwise (Koch, 2004).

5 Please not that Metzinger‘s call for a moratorium is quite different from a more recent call for a six month moratorium on the development of large language models earlier this year, which focused not on artificial suffering but on „profound risks to society and humanity“ (Ienca, 2023; Multiple, n.d.).

## Declarations

### Statements and Declarations

This manuscript represents original work not published elsewhere in any form or language.
